# The effect of hydrophobic chains on retarding performance of retarding acids

**DOI:** 10.1039/d2ra00552b

**Published:** 2022-03-22

**Authors:** Quan Hongping, Zhen Xuele, Lu Qiangying, Wang Linyuan, Jiang Silong

**Affiliations:** Oil & Gas Field Applied Chemistry Key Laboratory of Sichuan Province Chengdu 610500 P. R. China 59183228@qq.com +86-28-83037309; College of Chemistry and Chemical Engineering, Southwest Petroleum University Chengdu 610500 P. R. China

## Abstract

Acidizing retarders are used to reduce the reaction rate between hydrochloric acid (HCl) and carbonates to increase the efficiency of acidification of oil and gas wells and increase oil and gas recovery. We synthesized two polymeric acidizing retarders (AR-1 and AR-2) to explore ways to improve the efficiency of this process. Retarder AR-1 is based on acrylamide (AM) and allyl polyethylene glycol (APEG-1000), and retarder AR-2 is composed of AM, APEG-1000, and octadecyl dimethyl allyl ammonium chloride (C-18). The molecular structures of AR-1 and AR-2 were characterized by Fourier transform infrared (FT-IR) and ^1^H nuclear magnetic resonance (^1^H-NMR). The retarding performance and acid–rock reaction rates of AR-1 and AR-2 were evaluated, and the experimental results indicated that AR-2 had a better retarding performance than AR-1, and the acid–rock reaction rate of AR-1 was higher than AR-2. This could be because AR-2 with its hydrophobic chains forms a thicker adsorbed film. This was confirmed by X-ray photoelectron spectroscopy (XPS) and adsorption behavior studies. The scanning electron microscope (SEM) images, contact angle, and XPS results showed that both retarders form adsorption films on the surface of rocks, indicating that the introduction of AM and APEG-1000 makes the retarder adsorb on rock surfaces. The introduction of hydrophobic chains for AR-2 enhanced the thickness of adsorbed film, indicating that adding hydrophobic chains to the acidizing retarder significantly improves its retarding performance.

## Abstract 2

Acidizing retarders are used to reduce the reaction rate between hydrochloric acid (HCl) and carbonates to increase the efficiency of acidification of oil and gas wells and increase oil and gas recovery.

## Graphical abstract

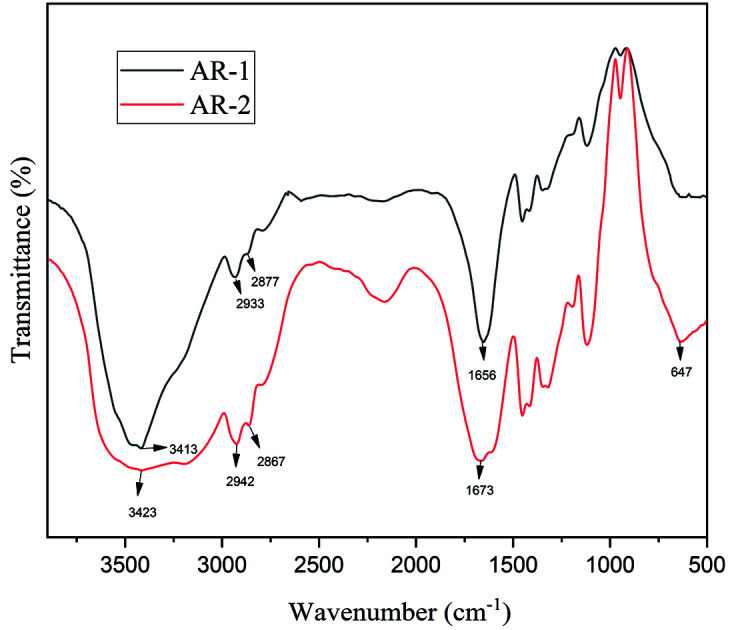

## 1. Introduction

Because of the world's continuing reliance on oil and natural gas, there has been concern about the decrease in the recovery rates of oil fields.^1–5^ Acidification, most commonly with hydrochloric acid (HCl), effectively increases the production of oil and gas wells,^6,7^ however, the efficiency of acidification is restricted because HCl cannot penetrate to the deeper places of reservoirs due to the fast reaction rate between the HCl and carbonates. Therefore, there has been a search for chemicals that could be added to the HCl to reduce the reaction rate between the HCl and carbonates and allow the HCl to penetrate more deeply into the reservoir.

In the past, thickening acid,^8^ foam acid,^9^ diverting acid,^10–13^ and others have been used to reduce the acid–rock reaction rate.^14–16^ The high viscosity of thickening acid effectively reduces the diffusion rate of hydrogen ions to the rock surface, thus reducing the reaction rate of acid–rock. However, the secondary damage caused by the thickening acid is a problem, and it is difficult to pump it in and out, so its application is limited. Foaming acid is a system with a mixture of acids, such as hydrochloric acid, hydrofluoric acid, and formic acid, introduced with bubbles created by adding a water-soluble polymer as a foaming agent and a foam stabilizer to the acid solution. Foaming acid with its high apparent viscosity and small filtration loss can effectively slow down the acid–rock reaction, but its foam stability is poor under high temperature conditions.^17^ The mixture of a chemical diverting agent and acid is called diverting acid. The existing diversion methods include *in situ* cross-linked acid and self-diverting acid.^18,19^ The diverting acid blocks some of the channels produced by dissolution, changing the flow profile and forcing the acid solution to flow into a relatively low permeability formation, which improves the acidification effect, but it is expensive and has poor temperature resistance and a complex construction technology.

At present, the retarding mechanisms are divided into two types. The first, reduces the diffusion rate of the H^+^ ions by increasing the viscosity of acids, the other separates the acid solution from the rock by forming a film on the surface of the rock, thus reducing the reaction rate between the acid and rock.

The adsorptive acidizing retarders include small molecular surfactants and macromolecular polymers synthesized using acrylamide. However, the former has the shortcomings of large dosage and high price, the latter mainly reduces the acid–rock reaction through the method of adsorbed film. Quan's^20–22^ research group proposed that a retarding acid with low viscosity could be adsorbed on the surface of carbonate rock and form a hydrated film that would delay the acid–rock reaction, but no published research has described a study of which factors could influence the film-forming properties.

In this paper, we describe our development of two low viscosity acidizing retarders (AR-1 and AR-2) that differ with respect to the presence of hydrophobic functional groups. AR-1 was synthesized using AM and APEG-1000, and AR-2 was synthesized using AM, APEG-1000, and C-18. The addition of C-18 adds hydrophobic groups to AR-2. We compared the retarding performance of the two acidizing retarders to better understand the key role of hydrophobic chains in retarding performance.

## 2. Materials and methods

### 2.1 Materials

Acrylamide (AM, AR), allyl polyethylene glycol (APEG-1000, >99%), hydrochloric acid (HCl, 37%), anhydrous ethanol (>99.5%), and potassium bromide (KBr, AR) were purchased from the Chengdu Kelong Chemical Factory. Octadecyl dimethyl allyl ammonium chloride (C-18, 70%) was bought from Jiangsu Fumiao chemical reagent plant. 2,2′-Azobis(2-methyl-propionamidine)dihydrochloride (V50, 97%) was purchased from Aladdin chemicals.

### 2.2 Synthesis of AR-1 and AR-2

The two reactions were completed in 250 mL three-necked flasks. We synthesized AR-1 by combining AM and APEG-1000 in a mole ratio of 24 : 0.25, and we synthesized AR-2 by combining AM, APEG-1000, and C-18 in a mole ratio of 22 : 1 : 1. We added V50 as an initiator. The two reactions were conducted at 50 °C for 5 h, and the two products were purified with ethanol and dried at 40 °C for 24 h.

### 2.3 Characterization and measurements

#### 2.3.1 Spectroscopic characterization

We generated FT-IR spectra of AR-1 and AR-2 using a WQF-520 Fourier transform infrared spectrophotometer, and we generated ^1^H-NMR spectra of AR-1 and AR-2 in D_2_O using a Bruker ASCENT-400 NMR (Switzerland) at room temperature.

#### 2.3.2 Gel permeation chromatography

We determined the molecular weights of AR-1 and AR-2 using gel permeation chromatography (GPC, PL-GPC50, USA).

#### 2.3.3 Scanning electron microscopy

We allowed the HCl in AR-1 and AR-2 to react with carbonate rock for 45 min at 50 °C, and we observed the morphology of the rock surface using scanning electron microscopy (SEM; JSM7500F, Japan).

#### 2.3.4 Contact angle analysis

We prepared AR-1 and AR-2 with different mass concentrations and reacted them with carbonate rock for 10 minutes at 50 °C. We then put water droplets on the rock surface and determined the contact angle of the rock surface using an interface parameter measuring instrument (KRUSS DSA30S, Germany).

#### 2.3.5 Retarding capability test of AR-1 and AR-2

We added AR-1 and AR-2 to HCl solutions to yield mass concentrations of 20%. The reactions between HCl solutions with the AR-1 and AR-2 added and carbonate rock were carried out at 30 °C, 50 °C, and 70 °C, with a rock surface area of 5 cm^2^. We calculated the retarding rate using eqn (1)–(3). Eqn (1) is

1 Δ*m = m*_1_ − *m*_2_

where Δ*m* is the dissolution mass of the rock sample (g), *m*_1_ is the mass of the rock sample before the reaction (g), and *m*_2_ is the mass of the rock sample after the reaction (g). eqn (2) and (3) are2
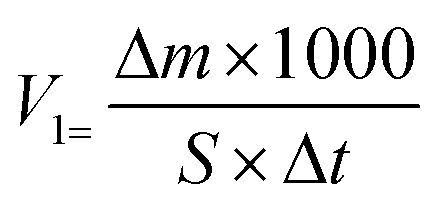
3
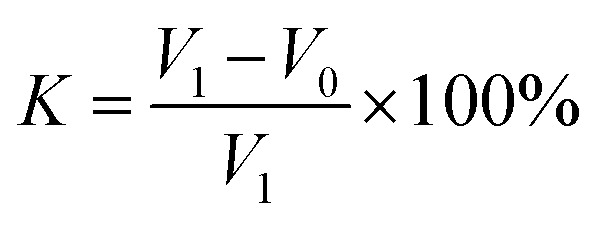
where *s* is the surface area of rock (cm^2^), Δ*t* is the reaction time (s), *K* is the retarding rate (%), *V*_0_ is the reaction rate of the AR-1 or AR-2 acid and rock (mg (cm^2^ s^−1^)^−1^), and *V*_1_ is the reaction rate of the hydrochloric acid without AR-1 and AR-2 and rock, (mg (cm^2^ s^−1^)^−1^).

#### 2.3.6 Determination of the reaction rates

We added different amounts of AR-1 and AR-2 to 20 wt% HCl to obtain different concentrations of AR-1 and AR-2 retarding acid solutions. We combined these solutions with carbonate rock at 50 °C and collected the CO_2_ gas produced by the reaction of the retarding acid and carbonate rock with a gas-collecting device. The acid–rock reaction rate was calculated using the volume of the collected CO_2_,^23,24^ and we calculated the reaction rate using eqn (4).4
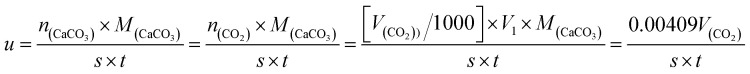
where *u* is the reaction rate of the retarding acid and carbonate rock (g cm^−2^ min^−1^), *n*_(CaCO_3_)_ is the amount of substance of CaCO_3_ (mol), *M*_(CaCO_3_)_ is the molar mass of CaCO_3_ in g mol^−1^, *S* is 5 cm^2^, *t* is the reaction time (min), *n*_(CO_2_)_ is the amount of substance of CO_2_ in mol, *V*_(CO_2_)_ is the volume of CO_2_ gas per unit of reaction time (mL), *V*_1_ is the molar volume at 25 °C.

## 3. Result and discussion

### 3.1 Characterization of AR-1 and AR-2

**Fig. 1:**
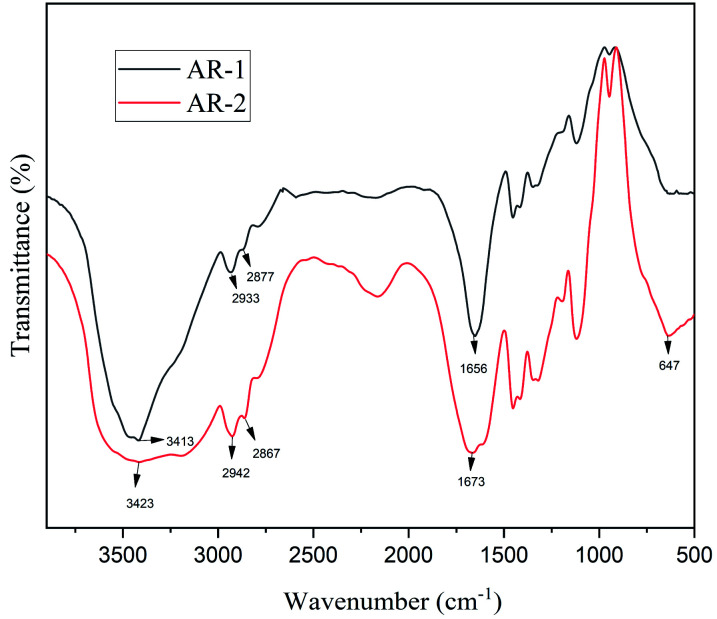
The FT-IR spectra of AR-1 and AR-2.

Fig. 1 shows the FT-IR spectra of AR-1 and AR-2. The peak at 3413 cm^−1^ for AR-1 corresponds to its O–H stretching vibration, and the peak at 3423 cm^−1^ for AR-2 corresponds to its O–H stretching vibration. The peaks at 2933 and 2877 cm^−1^ for AR-1 correspond to the C–H stretching vibrations of its –CH_2_ and –CH_3_ groups, and the peaks at 2942 cm^−1^ and 2867 cm^−1^ for AR-2 correspond to the C–H stretching vibrations of its –CH_2_ and –CH_3_ groups. Characteristic absorption bands at 1656 cm^−1^ (AR-1) and 1673 cm^−1^ (AR-2) correspond to C–O stretching vibrations of polyacrylamide, and the peak at 647 cm^−1^ for AR-2 correspond to the absorption peak of the C–H bonds of C-18. These results indicate that we successfully synthesized AR-1 and AR-2.

**Fig. 2:**
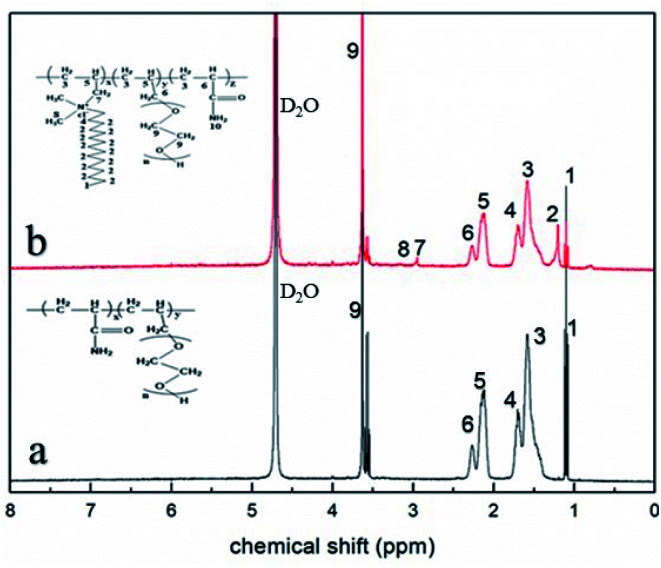
The ^1^H-NMR (400 MHz, D_2_O) spectra of (a) AR-1 and (b) AR-2.

Fig. 2 shows the ^1^H-NMR spectra of AR-1 and AR-2 that we used to provide further evidence of the successful synthesis of AR-1 and AR-2.

For AR-1, the chemical shift peaks of –CH_2_–CH– and –CH–(C=O)–) appear at 1.52 (marked as 3) and 2.20 ppm (marked as 6), respectively. The peak for –CH–CH_2_–O– is at 2.11 ppm (marked as 5). The peak at 3.59 ppm (marked as 9) is for –CH_2_–CH_2_–O–. The spectrum for AR-2 is similar to that of AR-1. The chemical shift peak for –CH–CH_2_–N– is at 2.11 ppm (marked as 5), and the characteristic chemical shift peaks for –CH_2_–N– and –N–CH_3_ appear at 3.28 (marked as 7) and 3.32 (marked as 8), respectively.

According to the results of our FT-IR and ^1^H-NMR analysis, we successfully synthesized AR-1 and AR-2, and their structures were consistent with our expected structures.

We measured the number-average molecular weight (*M*_n_) and the weight-average molecular weight (*M*_w_) of the two retarders, and Table 1 shows the results.

**Table 1:** Molecular characteristics of the two retarders^a^

Retarder	*M*_n_	*M*_w_	*M*_w_/*M*_n_
AR-1	120 106	568 777	4.74
AR-2	11 199	22 420	2.00

a The results showed that the average weight of two polymers were lower.

### 3.2 Carbonate rock sample analysis

**Fig. 3:**
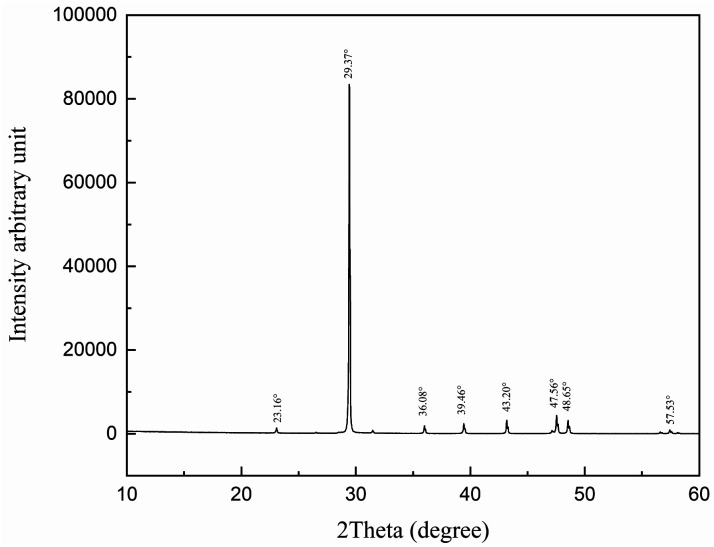
XRD patterns of the carbonate rock.

The purity of our experimental carbonate was determined by X-ray diffraction (XRD; X' Pert PRO MRD, Holland) with the XRD diffractogram recorded between 10° and 60° with Cu Kα X-rays at 85 W. Fig. 3 shows the results.

The interplanar spacing and the intensity of the diffraction peaks shown in Fig. 3 at 23.16°, 29.37°, 36.08°, 39.46°, 43.20°, 47.56° 48.65°, and 57.53° are consistent with CaCO_3_.^25^

### 3.3 The viscosity of the retarders

We added AR-1 and AR-2 to 20 wt% HCl to obtain different concentrations of samples, and we measured the viscosities of the mixtures. The viscosities were measured by a rotational viscometer (ZNN-D6, China) at 30 °C. Table 2 shows the results.

**Table 2:** Viscosities of AR-1 and AR-2 acids for different concentrations

Concentration (mg L^−1^)	The viscosity of AR-1 (mPa s)	The viscosity of AR-2 (mPa s)
2000	3	3
4000	3	3
6000	9	6
8000	18	15
10 000	27	18

The results in Table 2 show that although the viscosities were all low, increasing concentration of the mixtures led to a gradual increase in the viscosities of AR-1 and AR-2 acids. As we will demonstrate below, our experimental results showed that both AR-1 and AR-2 reduced the acid–rock reaction rate significantly despite their low viscosity, which is very different from the traditional method that requires high viscosity to reduce the acid–rock reaction rate.^26,27^

### 3.4 Effect of retarder dosage on retarding performance

**Fig. 4:**
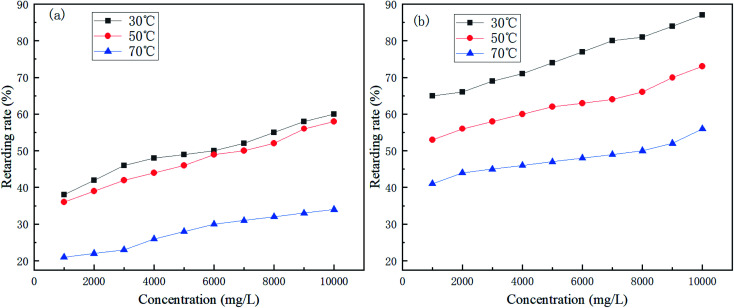
Variation of the retarding rate of (a) AR-1 and (b) AR-2 for different mass concentrations and different temperatures.

Fig. 4 shows the effect of retarder dosage and temperature on retarding performance.

Fig. 4 shows that the retarding rates of AR-1 and AR-2 decreased as the temperature increased at the same concentration, because the higher the temperature, the faster the acid–rock reaction rate. Fig. 4 also shows that the retarding rates increased gradually as the mass concentration increased at each temperature. A comparison of the retarding rates of AR-1 and AR-2 for the same mass concentration and same temperature shows that AR-2 had a significantly higher retarding rate than AR-1. Table 2 showed that when the concentrations of AR-1 and AR-2 were lower than 6000 mg L^−1^, their viscosities were the same, but when the concentrations were greater than 6000 mg L^−1^, the viscosities of AR-1 were higher than for AR-2. Because AR-2 has higher retarding rates than AR-1 despite its lower viscosity, the retarding mechanism must be different from the traditional method for which higher viscosity leads to decreased acid–rock reaction. The results also suggested that the introduction of hydrophobic chains on AR-2 molecules played a significant role in retarding performance.

### 3.5 Determination of the acid rock reaction rates

**Fig. 5:**
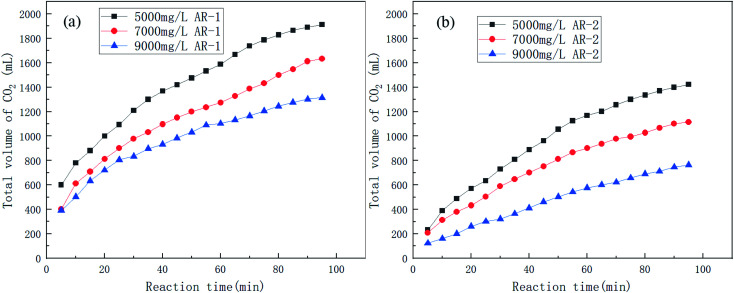
The volume of CO_2_ produced over time during the acid–rock rection for different concentrations of (a) AR-1 and (b) AR-2.

Fig. 5 shows the trend in CO_2_ production for the reaction of acid and carbonate for different concentrations of AR-1 and AR-2.

**Fig. 6:**
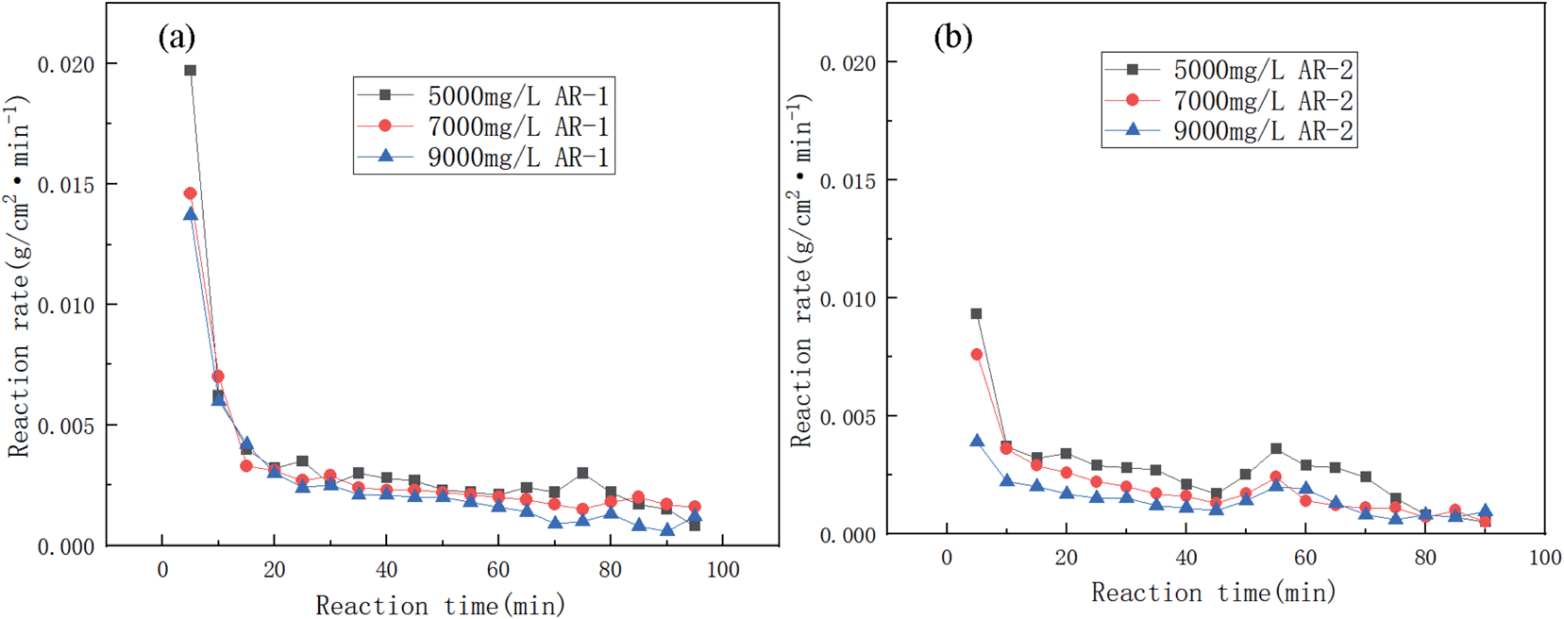
The reaction rates of the carbonate rock and retarding acid for different concentrations of (a) AR-1 and (b) AR-2.

Fig. 6 shows the trend in reaction rates for the reaction of acid and carbonate for different concentrations of AR-1 and AR-2.

Fig. 5 shows that the total volume of CO_2_ produced during the acid–rock reaction decreased with increasing polymer concentration. Fig. 6(a) shows that the reaction rate decreased with the increasing reaction time for all of the concentrations of AR-1, which could be due to the adsorption of the retarder AR-1 on the rock surface to form a film and the decrease of the HCl concentration. Fig. 6(b) shows that for all of the concentrations of AR-2, the reaction rate first decreased, then increased slightly, and finally decreased with increasing reaction time. The initial decrease in reaction rate could be due to the adsorption of the retarder AR-2 on the rock surface, forming a film that prevented the HCl from contacting the rock surface. The increase in reaction rate at about 45 min could be due to adsorption reaching saturation, followed by some desorption from the rock surface. Finally, the reaction rate gradually decreased due to a decrease of HCl concentration.

Comparison of the acid–rock reaction rate of AR-1 and AR-2 shows that the reaction rate of AR-1 was higher than for AR-2, indicating that the introduction of hydrophobic chains on AR-2 cause it to do a better job of blocking the reaction of HCl with the carbonate rocks. This will be explained below.

### 3.6 SEM analysis of the carbonate surface

**Fig. 7:**
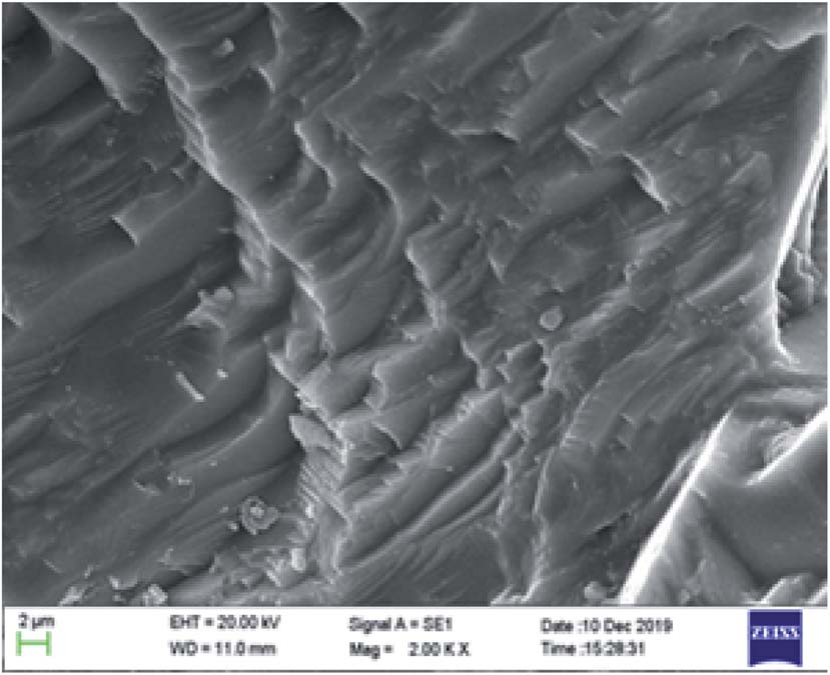
The SEM image of a carbonate sample treated with 20 wt% HCl (2000 times).

**Fig. 8:**
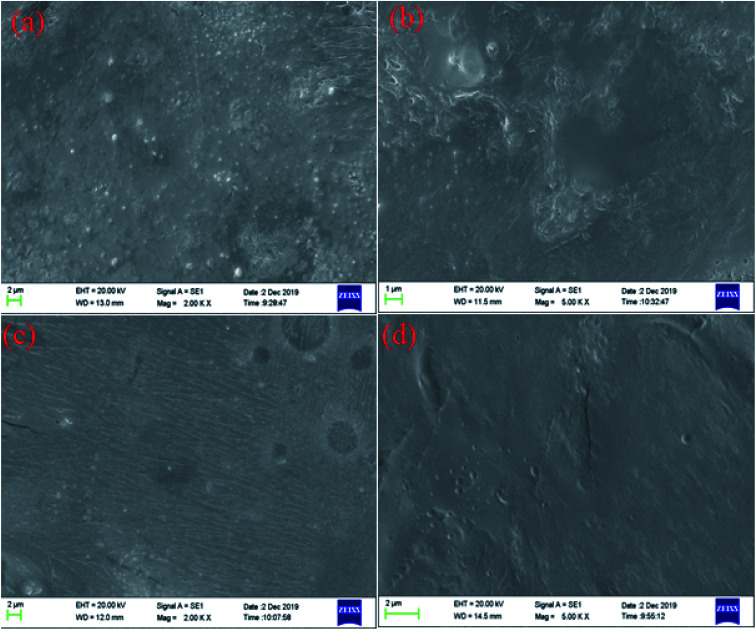
The SEM images of carbonate samples treated with (a) the AR-1 acid reacted with rock (2000 times), (b) the AR-1 acid reacted with rock (5000 times), (c) the AR-2 acid reacted with rock (2000 times), and (d) the AR-2 acid reacted with rock (5000 times).

The microstructure of carbonate rocks treated in different ways was obtained by SEM. Fig. 7 showed the SEM image of a carbonate sample treated with 20 wt% HCl, Fig. 8 shows the SEM images of carbonate samples treated with AR-1 and AR-2 acids.

Fig. 7 shows that the carbonate sample treated by 20 wt% HCl had an uneven and rough surface. Fig. 8(a) and (b) show that the carbonate sample that was treated by AR-1 acid looked relatively smooth, indicating the AR-1 formed a thin-layer of adsorbed film on the carbonate surface, and the Fig. 8(c) and (d) show that the rock surface that was treated by AR-2 acid was smoother and the film-forming properties were more obvious. This shows that both AR-1 and AR-2 can be absorbed on a carbonate rock surface to form an adsorption film. The adsorbed film prevented H^+^ ions from coming into contact with the rock surface, thus reducing the reaction rate between the acid and rock. The smoother and more obvious film shown in the SEM images for AR-2 helps to explain why AR-2 acts as a more efficient retarder.

### 3.7 The adsorption behavior of AR-1 and AR-2

Adsorption isotherms describe the relationship between the adsorption capacity of adsorbent and the equilibrium concentration of adsorbate in the liquid when the adsorption achieves equilibrium at a given temperature.^28^Eqn (5) is the equation for adsorption capacity,5
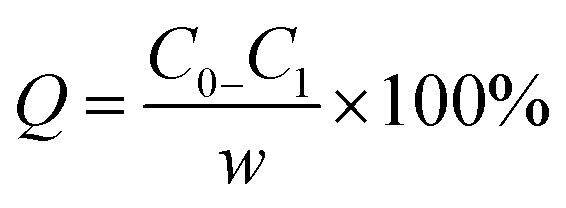
where *Q* is the adsorption capacity, mg g^−1^; *C*_0_ is the concentration before adsorption, mg L^−1^; *C*_1_ is the concentration after adsorption, mg L^−1^; *w* is the concentration of adsorbent, g L^−1^.

**Fig. 9:**
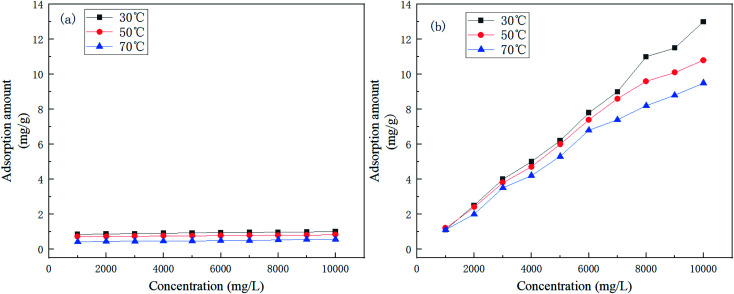
The adsorption isotherms of (a) AR-1 and (b) AR-2.

Fig. 9 shows the adsorption isotherms of AR-1 and AR-2 at 30 °C, 50 °C, and 70 °C.

Fig. 9 shows that at any concentration, the adsorption amount of AR-1 was lower than AR-2, indicating that the adsorption film of AR-2 adsorbed on a rock surface was thicker than AR-1, so AR-2 should be better at separating the acid solution from the rock. This could also explain why AR-2 has a better retarding performance than AR-1.

We further explored the adsorbing behavior and mechanism by fitting the data using Langmuir and Freundlich models. The Langmuir and Freundlich isotherm equations were expressed by eqn (6) and (7).^29^ The two models are most commonly used to determine isotherms for different adsorbent/adsorbate systems, to interpret solid–liquid adsorption, and predict their equilibrium parameters.6
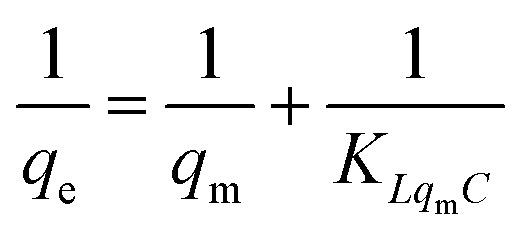
7
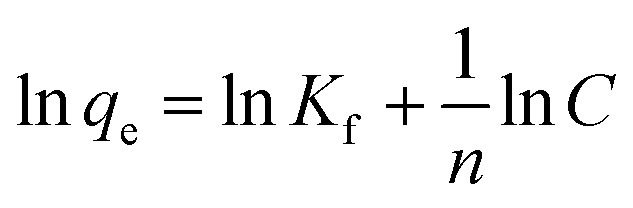
where *C* is the equilibrium concentration (mg L^−1^), *q*_e_ (mg g^−1^) and *q*_m_ (mg g^−1^) are the equilibrium and maximum adsorption capacity, respectively. The *K*_L_ (L mg^−1^) is the Langmuir coefficient and the *K*_f_ (mg g^−1^) is the Freundlich coefficient. The “*n*” parameter indicates the adsorption intensity.

Table 3 shows the isotherm constants for AR-1 and AR-2.

**Table 3:** Isotherm constants of retarders

	*T* (°C)	Langmuir			Freundlich		
		*Q*_max_ (μg mg^−1^)	*K*_L_	*R* ^2^	*K*_F_	*n*	*R* ^2^
AR-1	30	0.983	0.0033	0.9982	0.6139	21.0526	0.8603
	50	0.829	0.0029	0.9977	0.4949	19.3798	0.8208
	70	0.511	0.0028	0.998	0.2933	17.889	0.8563
AR-2	30	5000	2.20 × 10^−7^	0.0004	0.0011	0.996	0.9969
	50	68.49	2.08 × 10^−5^	0.8031	0.0022	1.0666	0.9964
	70	208.33	6.83 × 10^−6^	0.3119	0.0017	1.0295	0.9984

The Langmuir model yields the homogeneous adsorptive energies on the adsorbent surface. Table 3 shows that the correlation coefficient (*R*^2^) for AR-1 was close to 1 for the Langmuir model, indicating that the Langmuir model was appropriate for generating the isotherm adsorption data of AR-1. Because the polyoxyethylene ether chain at AR-1 helps AR-1 to be attracted to a carbonate surface by hydrogen bonds, the adsorption tended to be monolayer adsorption.

The Freundlich isotherm model yields the heterogeneous adsorptive energies on the adsorbent surface.^30^Table 3 shows that the correlation coefficient (*R*^2^) for AR-2 was close to 1, indicating that the Freundlich model was suitable for generating the isotherm adsorption data of AR-2. Because its hydrophobic chain is pointed away from the carbonate rock when AR-2 is adsorbed on the rock surface, and the association between the hydrophobic chains leads to multi-layer adsorption.

The difference of adsorption isotherm models between AR-1 and AR-2 demonstrates that the introduction of hydrophobic chains enhanced the thickness of adsorption film, which can further explain why the retarding effect of AR-2 is superior to AR-1.

### 3.8 The contact angle analysis

**Fig. 10:**
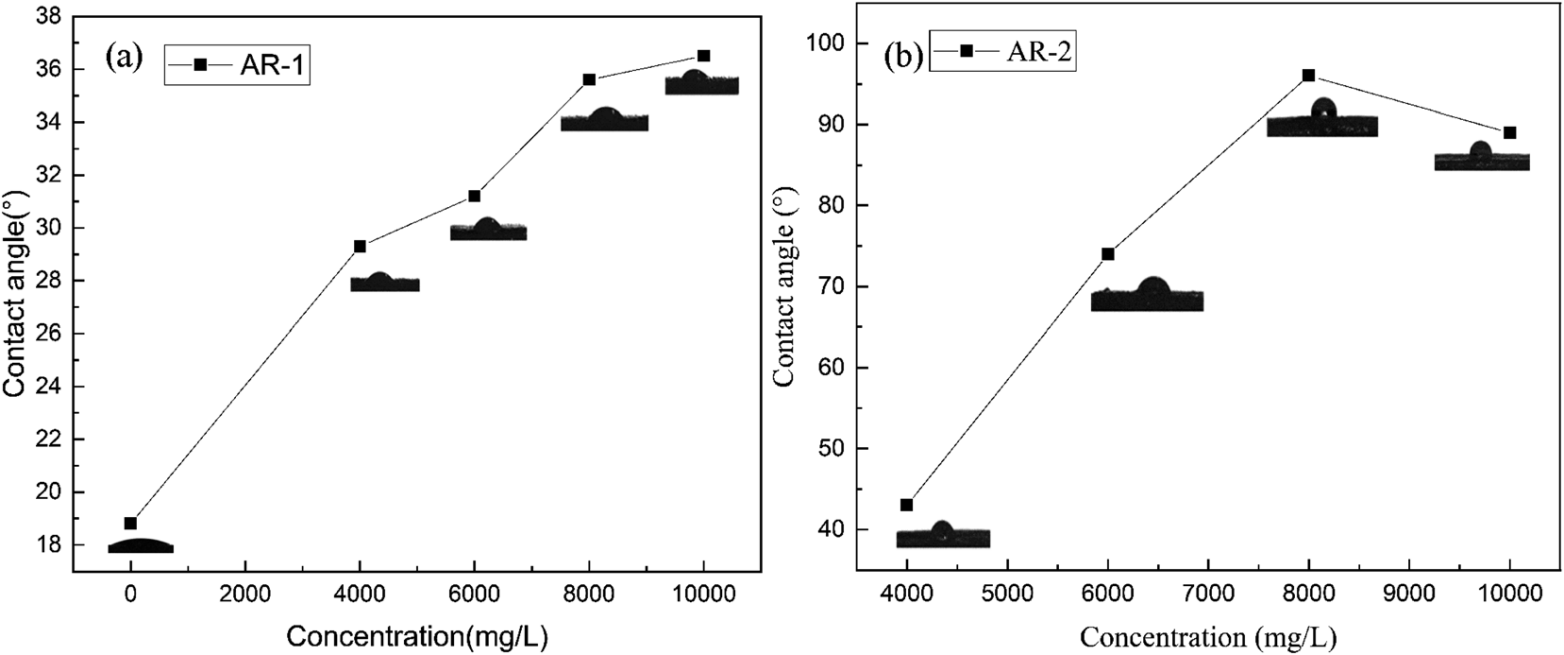
Contact angles for different mass concentration of (a) AR-1 and (b) AR-2.

We used contact angle to test the wettability of the rock surface.^31,32^Fig. 10 show the contact angles of rock surfaces for different mass concentrations of AR-1 and AR-2.

Fig. 10(a) shows that for AR-1, as the concentration increased, the contact angle increased, indicating a decrease in the hydrophilicity of the rock, but it was still less than 90 degrees, which is same as the water wetness of untreated rock.

Fig. 10(b) shows that when the concentration of AR-2 was less than 8000 mg L^−1^, the contact angle of the rock surface gradually increased with increasing concentration, but still was less than 90 degrees. This was because during the adsorption process of the AR-2, the hydrophobic chains of AR-2 were outward, which gradually changed the rock surface from hydrophilic to more hydrophobic. When the concentration of AR-2 was beyond 8000 mg L^−1^, the contact angle on the rock surface was greater than 90°, which could be due to the intermolecular association of polymers, and with the number of hydrophobic chains decreased, the rock surface was identified as water wetness gradually.

**Fig. 11:**
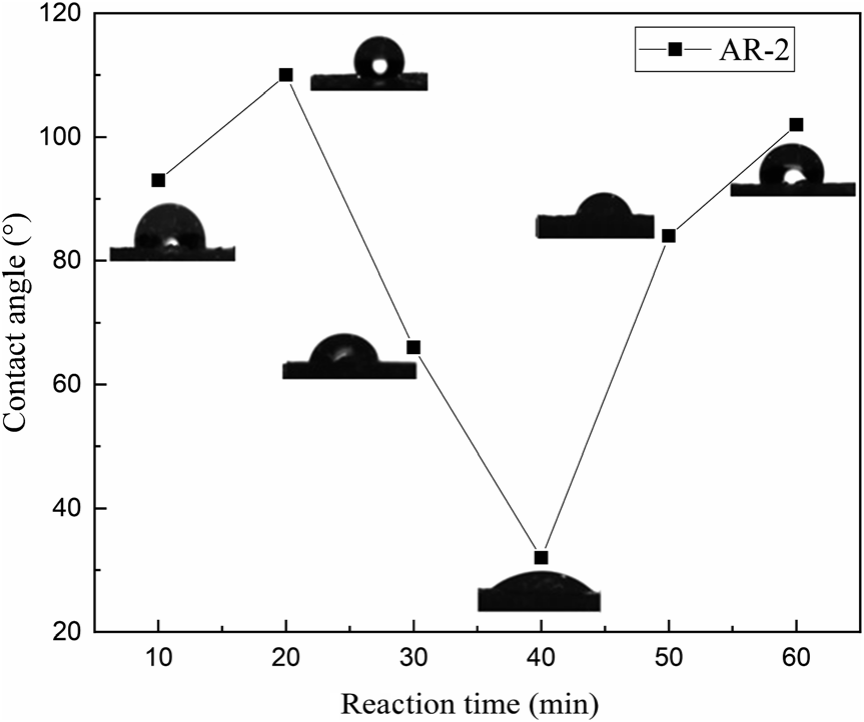
Contact angles for 8000 mg L^−1^ AR-2 with different reaction times.

To further understand the wettability of the rock surface after AR-2 acid interacted with it, we measured the contact angles of 8000 mg L^−1^ AR-2 for different acid–rock reaction times, and Fig. 11 shows the results.

Fig. 11 shows that as the reaction time of AR-2 increased, the contact angle increased at first and then decreased and then increased again, indicating that the wettability of the carbonate rock changed from increasing hydrophobicity to increasing hydrophilicity and then to increasing hydrophobicity again. The untreated rock surface is hydrophilic, so the increasing hydrophobicity shows that the retarder AR-2 was adsorbed on the rock surface, and the hydrophobic ends of the AR-2 were outward. As the reaction time increased, the wettability changed from increasing hydrophobicity to increasing hydrophilicity. A possible reason is that multilayer of AR-2 was added to the rock surface with its hydrophobic side interacting with the hydrophobic side, resulting in the hydrophilic side of the multilayer pointing away from the rock.^33–36^ When the reaction time was longer than 40 min, the wettability gradually changed from increasing hydrophilicity to increasing hydrophobicity. This could be due to desorption of the AR-2 from the rock surface, exposing the hydrophobic end of AR-2 on the rock surface. These trends provide evidence that there was multilayer adsorption of AR-2 on the rock surface.

In summary, the contact angle experimental results showed that the rock surface itself was hydrophilicity. Although the addition of the retarder AR-1 decreased the hydrophilicity of the rock, the rock surface remained hydrophilic. The adsorption of the retarder AR-2 on the rock surface led to a more significant decrease in the hydrophilicity of the rock surface with increasing AR-2 concentration and acid–rock reaction time, indicating that the introduction of a hydrophobic chain improved the film-forming ability of the AR-2 on the rock surface, making AR-2 a better retarder.

### 3.9 X-ray photoelectron spectroscopy analysis

**Fig. 12:**
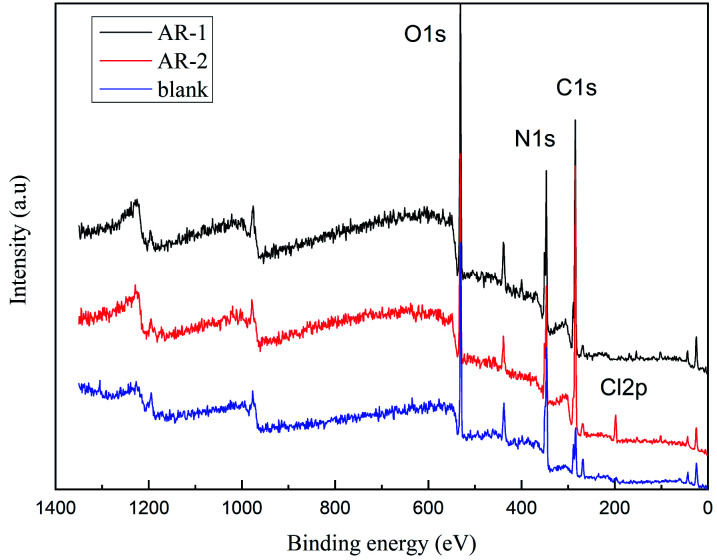
Results of X-ray photoelectron spectroscopy.

We did X-ray photoelectron spectroscopy (XPS, Escalab 250Xi England) analysis to determine the elemental composition of the substances^37,38^ on the rock surface after retarding acid interacted with rock. Fig. 12 shows the XPS analysis of C, N, O, and Cl for rock without AR-1 or AR-2, rock with AR-1, and rock with AR-2. The elements for untreated rock were different from the rock with the two retarding acids, indicating that both retarders had adsorbed on the rock surface. One difference between AR-1 and AR-2 is the presence of chlorine, Cl, in AR-2, and the XPS analysis showed Cl, indicating that AR-2 with its hydrophobic chains adsorbed on the rock surface.

We used eqn (8) and (9) to calculate the thickness of the adsorption film.8
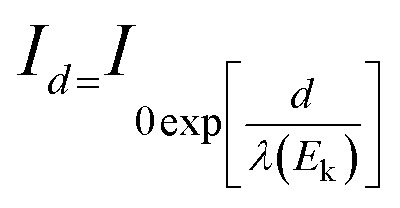

9 *λ*(*E*_K_) = 49*E*_k_^−2^ + 0.11(*E*_k_)^0.5^

where *I*_0_ and *I*_d_ are the initial and the thickness of the photoelectron intensity, *d* is the thickness of the adsorption layer, *E*_K_ is the optoelectronic kinetic energy, *λ*(*E*_K_) is the average escape depth of photoelectrons.

Table 4 shows the characteristic element optoelectronic test data.

**Table 4:** Characteristic element optoelectronic test data

Acid solution	*E*_K_ (eV)	*λ*(*E*_K_) (nm)	*I*_0_	*I*_d_	*d* (nm)
AR-1	1086.8	3.6264	0.3177	0.4854	1.5371
AR-2				0.5968	2.2864

Table 4 shows that the thicknesses of the AR-1 and AR-2 adsorbed films were 1.5371 nm and 2.2864 nm respectively, indicating that both retarders adsorbed on the rock surface to form an adsorption film, and the addition of hydrophobic chains of AR-2 increased the thickness of adsorbed film, thereby improving the retarding effect.

## 4. Conclusion

We synthesized two retarders, AR-1 and AR-2, and studied their retarding properties. The results showed that the retarding rate of AR-2 with its hydrophobic chain was higher than that of AR-1, and this was further verified by acid–rock reaction rate and SEM experiments. SEM images showed that there was an adsorption film on the rock surfaces treated by both retarding acids, and the thickness of the adsorption film was measured by XPS showed that the adsorption film thicknesses of AR-1 and AR-2 on the rock surface were 1.5371 nm and 2.2864 nm, respectively. The XPS and contact angle analysis of the rock surfaces after the acid–rock reactions verified that the two retarders were adsorbed on the rock surface. We found that the Langmuir model (monolayer adsorption) was a better fit for AR-1, and the Freundlich model (multi-layer adsorption) was a better fit for AR-2. This shows that the introduction of a hydrophobic chain on AR-2 increases the thickness of the adsorption films on rock surfaces, thereby improving the retarding rate of the retarding acid. This provides a direction for further research on retarding acids.

## Conflicts of interest

The authors declare that they have no known competing financial interests or personal relationships that could have appeared to influence the work reported on this paper.

## Supplementary Material
